# Natural History of Aortic Regurgitation following Percutaneous Mitral Valvuloplasty

**DOI:** 10.5812/cardiovascmed.8051

**Published:** 2013-02-24

**Authors:** Mohammadali Sadr-Ameli, Mona Heidarali, Sedigheh Saedi, Tehereh Saedi, Ata Firoozi, Mohsen Madani, Hooman Bakhshandeh

**Affiliations:** 1 Cardiac Electrophysiology Research Center, Rajaie Cardiovascular Medical and Research Center, Tehran University of Medical Sciences, Tehran, IR Iran; 2 Cardiovascular Intervention Research Center, Rajaie Cardiovascular Medical and Research Center, Tehran University of Medical Sciences, Tehran, IR Iran

**Keywords:** Percutaneous, Balloon Dilation, Mitral Valve Stenosis, Aortic Valve Insufficiency

## Abstract

**Background:**

Little is known about the natural history of aortic regurgitation (AR) in patients undergoing mitral valve procedures for mitral stenosis.

**Objectives:**

The aim of this study was to evaluate the short- and long-term effects of percutaneous mitral valvuloplasty (PMV) on coexisting AR.

**Materials and Methods:**

A total of 327 patients with rheumatic mitral stenosis (282 females and 45 males; mean age at the time of intervention = 47 ± 11 years) were followed up for between 48 hours and 13 years after PMV. At the time of PMV, 142 (43.3%) patients had no AR, 124 (37.9%) had mild AR, and 61 (18.7%) had moderate AR. After PMV, the follow-up showed that 120 (36.6%) patients had no AR, 103 (31.5%) had mild AR, and 104 (31.8%) had moderate AR.

**Results:**

AR progression after PMV and during the follow-up was significant (P < 0.00), but there was no significant increase in aortic valve replacement (AVR) procedures. The rate of AVR was higher in the moderate AR group (3.8%). There were no significant changes in the left atrial size (LA) (P = 0.6), ejection fraction (EF) (P = 0.4), and rhythm (P = 0.4) before and after PMV, respectively.

**Conclusions:**

Our findings indicate that among patients with rheumatic mitral stenosis, a considerable number have concurrent AR. Concomitant AR at the time of PMV does not influence procedural success and is not associated with inferior outcomes. Rheumatic aortic insufficiency progresses slowly by nature, and patients with AR and mitral stenosis can safely tolerate PMV without the possibility of undergoing AVR in the near future. Patients with moderate degrees of AR remain good candidates for PMV.

## 1. Background

Percutaneous mitral valvuloplasty (PMV) was first described by Inoue et al. (1) and has since been widely used to treat mitral stenosis (MS) (2). PMV is recommended in patients with moderate to severe MS who are symptomatic or have pulmonary hypertension but no left atrial thrombus and a favorable valve morphology (3). About half of the patients with rheumatic MS have some degree of aortic regurgitation (AR) (4). However; it is not clear whether these patients need aortic valve (AV) intervention in short- or long-term follow-up. The presence of AR does not affect the success of PMV procedure or its outcome (4). To resolve this issue, we require a complete understanding of the natural history of AV disease before and after PMV. AR intervention in case of non-severe AR during MV surgery is not recommended (5). Most previous studies have evaluated patients with mild AR and very few have been done in patients with moderate AR (4) and altogether little is known on the natural history of AR in patients after MV intervention (6-8). Given the high prevalence of rheumatic MS in our country, younger age of the affected patients, and insufficient number of the studies performed in this issue in spite of the high rates of PMVs performed, we sought to evaluate the natural history of AR and the short- and long-term effects of PMV on AR progression in our tertiary care center.

## 2. Objectives

The aim of this study was to evaluate the short- and long-term effects of percutaneous mitral valvuloplasty (PMV) on coexisting AR.

## 3. Materials and Methods

This retrospective study included 328 patients who underwent PMV at a tertiary centre between 1994 and 2007. Patients who had data available regarding the degree of pre-PMV AR were included in the analysis. The clinical and echocardiographic data were acquired from hospital records. All the patients underwent transthoracic echocardiography (TTE) and transesophageal echocardiography (TEE), and atrial size, pulmonary artery pressure, presence of LA thrombus, and MR were recorded. Patients with moderate to severe MS who had a valve area < 1cm^2^/m ^2^ of the body surface area and suitable valve morphology (MV score < 9) with no or mild mitral regurgitation (MR) and no left atrial clot were selected for PMV. All the procedures were performed via the Inoue method.1 Balloons were selected according to patient’s height and body surface area. Hemodynamic parameters, left and right atrial pressures, and gradient changes were recorded during PMV. Mitral valve area, MR, AR, tricuspid regurgitation severity, LVEF, and left atrial diameter were measured via TTE and TEE before the procedure. All the patients underwent TTE 48 hours after PMV and during the follow-up. The EF was defined mostly through the eyeballing method and noting regional wall motion abnormalities and degree of endocardial thickening. The EF and MR before and after PMV were also assessed by ventriculography, which was conducted in most cases before and after PMV.

Color Doppler, effective regurgitation orifice area, vena contracta width and pressure half time methods were used to define the quality and quantity of AR; and based on these findings, AR was classified into four groups: none, mild, moderate, and severe AR (9). None of selected patients had aortic stenosis. Left atrial diameter was measured from 2D images. The patients’ heart rhythms, based on the recorded ECGs, were grouped into sinus rhythm or AF before and after PMV. Demographic and clinical variables, including age, sex, and distribution of the data according to age and sex were recorded. The institutional ethics committee approved the research protocol.

### 3.1. Statistical Analysis

The continuous variables are expressed as Mean ± SD, and the categorical variables are expressed as a percentage. The Student’s t-test was employed for the interval variables, Fisher exact test and chi-square test for the categorical variables, and Mann Whitney U test for the ordinal variables (for the relationships between AR and sex, AR and rhythm, AR and AVR, and AR and MVR). Additionally, the non-parametric test was utilized as Kruskal-Wallis (for the relationship between AR and EF, and AR and LA) correlation and one-way Anova (the relationship between age and AR). The Mac Nemar test was opted for form non-parametric methods for the nominal data (rhythm before and after PMV), and the Wilcoxon signed rank test was used to assess the relationship between AR before and after PMV. All the statistical tests were two-sided using α < 0.05 level of significance. The data were managed and analyzed using SPSS 15 for windows (SPSS inc., Chicago, Illinois). For the confounding factors, repeated measured ANOVA and multiple linear regressions were used. The LV diameters were measured using the paired sample corrections, student t-test, and Friedman test. Data were collected using a personal code for each patient.

## 4. Results

A total of 328 patients underwent PMV preceded by echocardiography during a 13-year period from 1997 to 2010 in a tertiary cardiovascular center. The study population consisted of 282 (86.3%) women and 45 (13.7%) men. A pregnant woman who suffered from critically severe MS and severe AR and underwent successful PMV and accomplished her pregnancy but refused the follow-up process was excluded. The patients were divided into three groups: no AR, mild AR, and moderate AR. The mean age of our patients was 47.16 ± 11 years (Table 1). AR was measured before the procedure via echocardiography; and of a total of 327 patients, 142 (43.2%) had no AR, 124 (37.9%) had mild AR, and 61(18.7%) had moderate AR. The relationship between age and AR severity was not statistically significant (P = 0.7), and nor was there a significant relationship between sex and AR (P = 0.8). The mean of the EF was 52.5 ± 4.3. The EF and AR had a significant relationship (P = 0.02).There was also a significant relationship between the EF and rhythm (P = 0.01), and the patients with sinus rhythm showed a higher mean of EF (52.9 ± 4.1 vs. 51.3 ± 4.9). The relationship between sex and rhythm was not significant (P = 0.7) (Table 1). There was no significant relationship between rhythm and AR severity before PMV (P = 0.2). The pre-procedural characteristics of the study population are shown in Table 1.

**Table 1.** Baseline characteristic grouped according to severity of pre-valvuloplasty aortic regurgitation AR n= 327[Table-fn fn165]

	AR a			
	No (n = 142)	Mild (n = 124)	Moderate (n = 61)	*P *Value
**Female**	122 (43.3%)	109 (38.7%)	51 (18.1%)	0.8
**Age, y**	46.6 ± 11.1	47.4 ± 11.4	47.7 ± 9.5	0.7
**AF a**	35.4%	41.8%	22.6%	0.2
**Sinus rhythm**	45.9%	39.7%	14.4%	0.2
**EF a**	52.8 ± 3.8	51.7 ± 4.8	53.4 ± 4.1	0.02
**LA diameter**	4.5 ± 0.6	4.7 ± 0.5	4.5 ± 0.7	0.1

Values are mean ± SD or n (%)

^a^Abbreviations: AF: sinus rhythm or AF before and after PMV, AR: Aortic Regurgitation, EF: Ejection Fraction, LA: Left Atrial

Follow-up data showed that after PMV, 120 (36.6%) patients had no AR, 103 (31.5%) had mild AR, and 104 (31.8%) had moderate AR. Altogether, after PMV, 207(63%) patients had AR. Of the 142 patients, who had no AR before PMV, 120 patients remained the same during the thirteen years of follow-up and 22 patients progressed to mild AR. Forty three (34.6%) patients, who had mild AR initially, showed moderate AR, which was statistically significant. None of our patients had progressed to severe AR. AR progression been mostly prevalent in the women’s group. The follow-up results of the study are depicted in Table 2.

**Table 2.** Characteristics grouped after Percutaneous balloon mitral valvuloplasty (n = 327)[Table-fn fn167]

	AR a			
	No (n = 120)	Mild (n = 103)	Moderate (n = 104)	*P *Value
**Female**	104(36.9%)	91(32.3%)	87(30.9%)	0.5
**Age**	47.6 ± 10.7	48.3 ± 11.7	45.3 ± 10.3	0.1
**AF a**	34.2%	33.3%	32.4%	0.2
**Sinus rhythm**	39%	31.1%	29.9%	0.2
**EF a**	51.1 ± 5.0	51.4 ± 5.3	52.04 ± 4.5	0.4
**LA diameter**	4.6 ± 0.6	4.7 ± 0.6	4.5 ± 0.7	0.1

Values are mean ± SD or in (%)

^a^ Abbreviations: AF: sinus rhythm or AF before and after PMV, AR: Aortic Regurgitation, EF: Ejection Fraction, Mod AR: Moderate AR, LA: Left Atrial Size

After PMV, the mean EF was 51% ± 5, which was reduced by 1.1%. After adjusting for age and sex as the confounding factors, PMV had no effect on the EF. There was no significant relationship between AR progression and the EF (P = 0.46). In our study there was no significant relationship between AR progression and rhythm changes after PMV (P = 0.4). Sinus rhythm was again more prevalent after PMV, but 32 (9%) of the patients developed AF. The mean LA diameter after PMV was 4.62 ± 0.64mm.The association between increasing age and the LA diameter was measured before and after PMV and was statistically significant (P = 0.001). Four (3.8%) out of the 104 patients with moderate AR after PMV underwent aortic valve replacement (AVR) and concomitant mitral valve replacement (MVR) for mitral valve disease severity as main indication. None of the patients with no or mild AR underwent AVR and no patient underwent sole AVR due to AR severity. There was no significant association between AR progression and the MVR procedure after PMV (P = 0.4) (Table 3).

**Table 3.** Prevalence of MVR based on severity of preexisting AR n=327[Table-fn fn170]

	AR a			
	No (n = 120)	Mild (n = 103)	Moderate (n = 104)	*P *Value
**MVR a**	7 (5.8%)	8 (7.7%)	9 (8.6%)	0.4
**AVR a**	0	0	4 (3.8%)	0.01

Values are mean ± SD or in (%)

^a^Abbreviations: AR: Aortic regurgitation, AVR: Aortic Valve replacement, MVR: Mitral Valve Replacement

## 5. Discussion

Rheumatic heart disease (RHD) continues to be a common health problem in the developing world, causing morbidity and mortality among children and adults (10). Concurrent involvement of both MV and AV has been reported in one third to one half of patients with RHD (9, 11, 12). We investigated the natural history of pre-existing AR after the PMV procedure. Some studies have postulated that relieving MS may increase the amount of AR, which may prove to be an intolerable burden on the LV or become so severe that AVR would be needed (13). After PMV for severe MS, one might expect AR to increase significantly, mostly due to enhanced blood flow through previously diseased AV and bring about the need for AVR at an earlier than expected time. On the other hand few other available natural history studies on this condition have not confirmed this (8, 12, 14). The present study demonstrated although a slow progression to mild or moderate AR was common, no significant progression to severe AR was recorded. In our study, more patients had moderate AR before PMV compared to previous studies. A few needed AV replacements during the follow-up but none of the patients required AV replacement due to AR progression as the sole indication. 

Patients with severe AR and concomitant MS are usually not referred for PMV. On the other hand, combined aortic and mitral valve replacement is usually associated with higher procedural risks and poorer long-term survival than is the replacement of either of the two valves (7). Based on this study, we think PMV could also be beneficial in patients with severe AR who do not yet serve the true indication of AVR. Future studies might better clarify this point. Our patients’ mean age at the time of MV intervention was 47.1 ±11 years, which was close to that in the study done by the Vaturis group (7, 8, 12). These results confirmed that the mean age of presentation for MV procedures in our country is midway between that in underdeveloped and developed countries (15).

Significant MS and resultant PMV were mostly seen in the women in the present study. The Numboodiri study and other studies also reported predominant patterns in females (4, 12). As stated previously in the results section, AR progression was more common in the women’s group which could be attributed to more prevalent and severe rheumatic heart involvement in females. Our older patients were more likely to have atrial fibrillation (AF) before and after PMV (54.2 ± 9.7and 52.7 + 9.8) respectively, and AF increased significantly according to age. These findings are similar to those in some other studies (4, 16). Our analysis confirmed that many patients had sinus rhythm before PMV and it was probably due to the selection of lower-risk patients in the earlier stages of MS for PMV. After PMV and during the follow-up period, sinus rhythm remained predominant in 61.5% patients but a considerable minority of the patients (7.5%) who had AF before PMV had converted sinus rhythm during the follow-up. This trend showed the possibility of reverse remodeling in the left atrial changes and conducting system if PMV is preformed early and in appropriate candidates. This group of patients was younger and had a smaller left atrial size, higher EF, and effective valve area after PMV. In patients who had AF rhythm, before and after PMV, the left atrial size was larger, which was correlated with poorer clinical outcome and valvular morphology (4, 16). 

The older patients had a larger left atrial size before PMV and the mean diameter was greater in the men. The diameter of the left atrium after PMV increased 1.4mm, which was not statistically significant. In our study after PMV, the mean diameter of the left atrium in the patients with sinus rhythm was decreased (2mm) but the mean diameter of the left atrium in the patients with AF was increased (2mm). Furthermore, a 1.1% reduction was seen in the EF of the patients during the follow-up in contradiction to our first hypothesis that the EF might increase due to improved preload. This may be in consequence of the increasing age, change of the rhythm, different degrees of AR, and baseline RHD. The mean of the EF before and after PMV was not significantly different between our men and women, but the EF reduction was more predominant after PMV in the females. 

Although there was no significant relationship between the patients’ rhythm and AR (P = 0.2, P = 0.4) before and after PMV, it seems that the patients without AR were more likely to have sinus rhythm, which may be due to milder forms of the disease. The patients who underwent MVR were mostly women and older, had larger left atrium and their rhythm was mostly AF. As pointed in results section, in no case was AVR performed as a single procedure. All the patients who underwent AVR had moderate AR before surgery.

### 5.1. Limitations

Most of the patients had mild or moderate AR in our study and there was no assessment of patients with severe AR. One patient with severe AR before PMV tolerated the procedure and her pregnancy well but we do not have long-term follow-up data. Our findings indicate that among patients with rheumatic MS and planned PMV, a considerable number have concomitant aortic regurgitation. However, progression of AR after PMV is slow and PMV does not increase the rate of AVR. Based on these results, we would suggest that PMV is an effective treatment for patients who have MS and concurrent trivial to moderate AR and is not associated with inferior outcomes.

## References

[1] Inoue K, Owaki T, Nakamura T, Kitamura F, Miyamoto N (1984). Clinical application of transvenous mitral commissurotomy by a new balloon catheter... J Thorac Cardiovasc Surg..

[2] Tops LF, Kapadia SR, Tuzcu EM, Vahanian A, Alfieri O, Webb JG (2008). Percutaneous valve procedures: an update.. Curr Probl Cardiol..

[3] Bonow RO, Carabello BA, Kanu C, de Leon AC, Jr., Faxon DP, Freed MD (2006). ACC/AHA 2006 guidelines for the management of patients with valvular heart disease: a report of the American College of Cardiology/American Heart Association Task Force on Practice Guidelines (writing committee to revise the 1998 Guidelines for the Management of Patients With Valvular Heart Disease): developed in collaboration with the Society of Cardiovascular Anesthesiologists: endorsed by the Society for Cardiovascular Angiography and Interventions and the Society of Thoracic Surgeons.. Circulation..

[4] Sanchez-Ledesma M, Cruz-Gonzalez I, Sanchez PL, Martin-Moreiras J, Jneid H, Rengifo-Moreno P (2008). Impact of concomitant aortic regurgitation on percutaneous mitral valvuloplasty: Immediate results, short-term, and long-term outcome.. Am Heart J..

[5] Bernal JM, Fernandez-Vals M, Rabasa JM, Gutierrez-Garcia F, Morales C, Revuelta JM (1998). Repair of nonsevere rheumatic aortic valve disease during other valvular procedures: is it safe?. J Thorac Cardiovasc Surg..

[6] Choudhary SK, Talwar S, Juneja R, Kumar AS (2001). Fate of mild aortic valve disease after mitral valve intervention.. J Thorac Cardiovasc Surg..

[7] Vaturi M, Porter A, Adler Y, Shapira Y, Sahar G, Vidne B (1999). The natural history of aortic valve disease after mitral valve surgery.. J Am Coll Cardiol..

[8] Ha JW, Choi SH, Chang BC, Nam CM, Jang Y, Chung N (2002). Is prophylactic aortic valve replacement indicated during mitral valve surgery for mild to moderate aortic valve disease?. Ann Thorac Surg..

[9] Spagnuolo M, Kloth H, Taranta A, Doyle E, Pasternack B (1971). Natural history of rheumatic aortic regurgitation. Criteria predictive of death, congestive heart failure, and angina in young patients.. Circulation..

[10] Eisenberg MJ (1993). Rheumatic heart disease in the developing world: prevalence, prevention, and control.. Eur Heart J..

[11] Bland EF, Duckett Jones T (1951). Rheumatic fever and rheumatic heart disease; a twenty year report on 1000 patients followed since childhood.. Circulation..

[12] Namboodiri N, Remash K, Tharakan JA, Shajeem O, Nair K, Titus T (2009). Natural history of aortic valve disease following intervention for rheumatic mitral valve disease.. J Heart Valve Dis..

[13] Runco V, Levin H, Wooley C, Booth R (1965). Aortic regurgitation: Angiography study of its natural history following mitral valvotomy and its effect on post operative prognosis.. Circulation..

[14] Padial LR, Oliver A, Vivaldi M, Sagie A, Freitas N, Weyman AE (1997). Doppler echocardiographic assessment of progression of aortic regurgitation.. Am J Cardiol..

[15] Libby P, Bonow RO, Mann DL, Zipes DP (2011). Braunwald's Heart Disease: A Textbook of Cardiovascular Medicine..

[16] Maatouk F, Betbout F, Ben-Farhat M, Addad F, Gamra H, Ben-Hamda K (2005). Balloon mitral commissurotomy for patients with mitral stenosis in atrial fibrillation: ten-year clinical and echocardiographic actuarial results.. J Heart Valve Dis..

